# Climate change or irrigated agriculture – what drives the water level decline of Lake Urmia

**DOI:** 10.1038/s41598-019-57150-y

**Published:** 2020-01-14

**Authors:** Stephan Schulz, Sahand Darehshouri, Elmira Hassanzadeh, Massoud Tajrishy, Christoph Schüth

**Affiliations:** 1 Technische Universität Darmstadt, Institute of Applied Geosciences, Schnittspahnstr. 9, 64287 Darmstadt, Germany; 2 Polytechnique Montréal – Department of Civil, Geological and Mining Engineering, Montreal, Canada; 3 Sharif University of Technology, Urmia Lake Restoration Program, Department of Civil Engineering, Azadi Ave, P.O.Box: 11155, 9313 Tehran, Iran

**Keywords:** Hydrology, Limnology

## Abstract

Lake Urmia is one of the largest hypersaline lakes on earth with a unique biodiversity. Over the past two decades the lake water level declined dramatically, threatening the functionality of the lake’s ecosystems. There is a controversial debate about the reasons for this decline, with either mismanagement of the water resources, or climatic changes assumed to be the main cause. In this study we quantified the water budget components of Lake Urmia and analyzed their temporal evolution and interplay over the last five decades. With this we can show that variations of Lake Urmia’s water level during the analyzed period were mainly triggered by climatic changes. However, under the current climatic conditions agricultural water extraction volumes are significant compared to the remaining surface water inflow volumes. Changes in agricultural water withdrawal would have a significant impact on the lake volume and could either stabilize the lake, or lead to its complete collapse.

## Introduction

**Figure 1:**
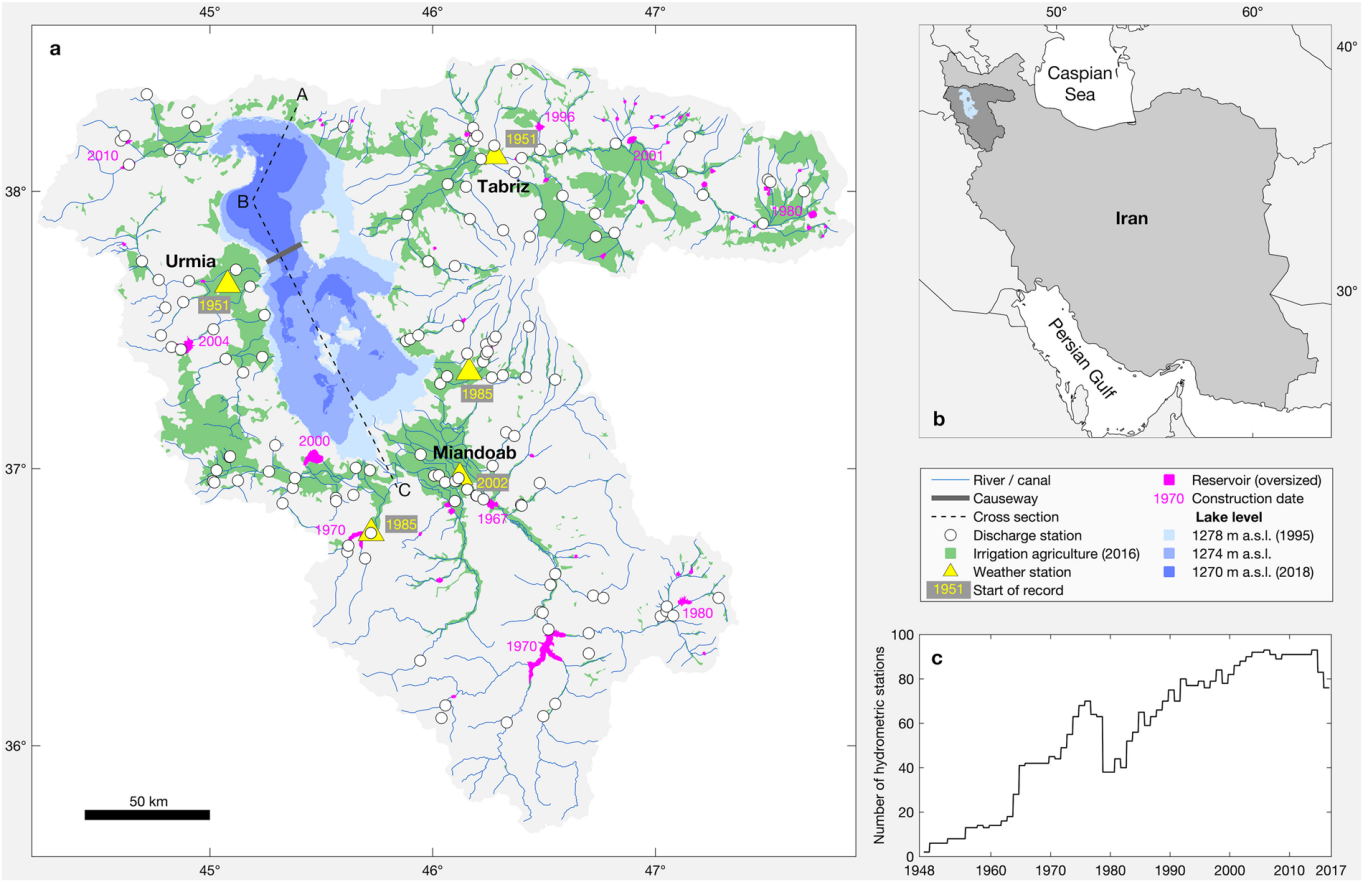
Geographic overview of Lake Urmia basin. (**a**) Location of monitoring stations, and minimum and maximum lake extent, and extent at lowest lake level (1274 m a.s.l.) before 1995. (**b**) Regional overview. (**c**) Number of hydrometric stations over time. (Plots and maps are generated using MATLAB R2019b, www.mathworks.com).

Lake Urmia is an endorheic lake located in north-west of Iran (Fig. 1). With an average original surface area of about 5,000 km^2^ it is one of the largest hypersaline lakes on earth^1–3^. Considering its original extent, Lake Urmia has more than one hundred islands, which are vital for the reproduction of various local birds, but also as a safe breeding refuge of migratory birds such as Flamingos and White Pelicans^2^. The main islands are an ideal habitat for endangered species such as the Iranian yellow deer and Armenian mouflon^4^. Despite its very high natural salinity of 140–280 gL^−1^ ^5,6^, the lake itself constitutes a living space for diverse bacterial communities, halophilic phytoplankton, or the brine shrimp *Artemia urmiana*^4^.

Starting in 1995, Lake Urmia experienced a strong decline in lake level. Between 1995 and 2013 the lake lost about 60% of area and even more than 90% of its volume^7–10^. Although the interannual variability of the lake level has always been high, the extreme decline in the 90 s is a singular event, at least in the last 100 years^11,12^. This loss of volume has negative impacts on the lake’s ecosystems, such as a significant reduction of the aquatic habitat accompanied by an increase of salinity to more than 300 gL^−1^ ^2^, which has caused a severe slowdown of the reproduction rate of *Artemia Urmiana*^13^. Moreover, most islands have disappeared and the deposition of sand dunes and evaporites on the dried-up lake bed has formed a vast salty desert.

The reasons behind the pronounced water level decline during the past two decades are controversial. Several studies state that the reduction of surface water inflow due to agricultural extraction predominantly caused the lake level decline^14–22^. This was mainly triggered by an uncontrolled growth of the irrigated area, accompanied by the extensive construction of reservoirs, and poor agricultural water use efficiency. Several studies base their conclusions on trend analysis of hydro-meteorological data sets^8,19,22,23^. While Jalili *et al*.^19^ and Khazaei *et al*.^22^ identified only weak correlations between meteorological variables and lake level and hence concluded that mainly anthropogenic changes caused the lake level decline, Alizadeh-Choobari *et al*.^8^ and Zoljoodi and Didevarasl^23^ could identify more pronounced trends in climate variables, i.e. increasing temperatures and decreasing precipitation over the last decades. Interestingly, the studies from the former authors base on rather large-scale satellite products, while the latter have used records of local weather stations. Three of the studies mentioned above even provide quantitative estimates of anthropogenic impacts. Ghale *et al*.^16^ concluded on the basis of a time series analysis comparing the water balance of the lake with agricultural water consumption that about 80% of the shrinkage in the period 1998–2010 was man-made. Using a hydrological model, Chaudhari *et al*.^15^ found that 86% of the shrinkage of the lake in the period 1995–2010 can be explained by human activities. Hassanzadeh *et al*.^17^ used a system dynamics model^24^ to analyze the influence of different management and climate scenarios on the lake volume. Their model results suggest that the influence of the reservoirs (at a storage volume of about 1 km^3^), the changes in precipitation directly above the lake, and the reduced inflow due to overuse of surface water are responsible for 25%, 10% and 65% of the volume loss, respectively.

Other studies conclude that changes in climate, i.e. reduced precipitation and increased temperatures, are mainly responsible for lowering the lake level^7,9,25–27^. Generally, these studies based their assumptions on similarities between trend patterns of climate and inflow into the lake. Fathian *et al*.^26^ showed that decreasing discharge trends can already be observed in the headwater catchment areas, which indicate climate-induced changes, and that the reservoirs do not seem to have a significant influence. Shadkam *et al*.^7^ analyzed the relative contribution of climate change and water management to the water balance of the lake using a variable infiltration capacity model. They also conclude that reservoirs have no significant impact on the reduced lake inflow and that climate and irrigation have an impact of 60% and 40% respectively. Furthermore, they state that the increased irrigation water demand results from stronger and longer periods of drought and thus establish a direct correlation between the increasing agricultural water demand and climatic changes.

The future of Lake Urmia is at stake. The relevance and interplay of global and regional factors, i.e. climate change and local water management, will determine its fate. Although our ability to influence climatic changes seems to be rather limited, at least on a short time scale, an optimization of agricultural practices has a proven potential for immediate water savings^28^. However, to encourage and justify related water savings requires a prediction of their potential benefits based on scientifically sound future projections. To this end, we quantify the components of the water budget of Lake Urmia over the last five decades and analyze their temporal evolution and interplay. Based on the water balance, we perform a series of simulations of different development scenarios to analyze whether and to what extend local agricultural water saving could contribute to the restoration and conservation of Lake Urmia.

## Results and Discussion

### Lake Urmia’s water balance

**Figure 2:**
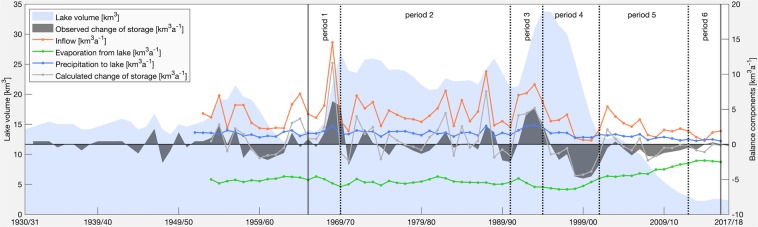
Water balance of Lake Urmia. Temporal evolution of Lake Urmia’s water balance components (seasonal sums) and observed lake volume (seasonal average). A hydrological season in the Lake Urmia basin extends from October to September of the following year. (Plot is generated using MATLAB R2019b, www.mathworks.com).

Lake Urmia is the limnic end member of an endorheic (closed) basin, which means that its only relevant outflow component is the evaporation (*E*) from the lake surface. Inflow into the lake results from several rivers (*Q*) and from direct precipitation (*P*). Also, a certain groundwater component in the water balance cannot be precluded, especially if one considers the groundwater extraction through tens of thousands of legal and illegal wells in the Lake Urmia catchment^29^. To the best of our knowledge, however, the vast majority of studies assume that a direct groundwater component is relatively small compared to the previously mentioned ones. For instance, hydrochemical investigations at the western shore indicated almost no direct hydraulic interaction between groundwater and lake water^30^, another study considered a direct component to be less than 3% of total inflow^7^, and some studies provide even quantitative estimates ranging from about 60 × 10^6^ m^3^a^−1^ to 210 × 10^6^ m^3^a^−1^ ^29,31,32^. The reason for these rather low rates is the fact that the main receiving water bodies for the groundwater are the perennial rivers discharging into the lake^1,7,32,33^. Therefore, a direct groundwater component is neglected in the water balance of the lake, Eq. (1).

1 $$\frac{dS}{dt}=\frac{P+Q-E}{dt}$$

where dS is the change of water storage in the lake over the time period dt. Available data sets allowed the computation of the water balance from October 1953 to September 2017 with hydrological seasons extending from October to September in the following year (Fig. 2).

Between 1965 and 1995 the lake received an annual average of 4.9 km^3^ water from its tributaries. Despite strong interannual variations, after 1995 a decline in river water inflow is evident, i.e. the mean annual discharge rate dropped by 50% to about 2.4 km^3^a^−1^ (1995 to 2017). The lowest annual surface discharge to the lake of only 0.5 km^3^ was recorded in 2015 (Fig. 2). The total volume of precipitation input and evaporation loss depend on the extent of the lake. Moreover, the actual evaporation rate depends on the salt concentration, which increases by reduction in lake volume. As a consequence of both, there has been a large evaporation rate in 1990s and the previous decades (up to 8.8 km^3^a^−1^). Thus, the decreasing evaporation loss to some extend buffers the lower inflow rates and leads to an almost equilibrated water balance since 2013. The calculated changes in the lake volume by considering the water balance components and the observed changes in lake volume agree quite well (Fig. 2), indicating the validity of the proposed water balance.

For statistical analysis, which is the subject of the next section, we distinguished six periods between 1965 and 2017 that are characterized by their general trends in the lake volume evolution. Although, the available data set allowed us to draw the water balance for the lake since 1953, the number of operating hydrometric stations is rather limited before 1965, i.e. they have more than doubled from 1963 (n = 18) to 1965 (n = 42, Fig. 1c), which likewise improves the robustness of the data set. Besides, the separation of the periods bases on a visual analysis of storage change patterns, i.e. steep increase in lake volume during periods 1 and 3 with an observed change of storage (dS) of 3.2 km^3^a^−1^ in both cases, a quite stable lake volume during periods 2 and 6 with dS = −0.3 km^3^a^−1^ and 0.1 km^3^a^−1^, respectively, a steep decrease in lake volume during period 4 (dS = −3.0 km^3^a^−1^), and a moderate decrease during period 5 (dS = −0.9 km^3^a^−1^, Fig. 2).

### Changing climate vs. irrigation water consumption

**Figure 3:**
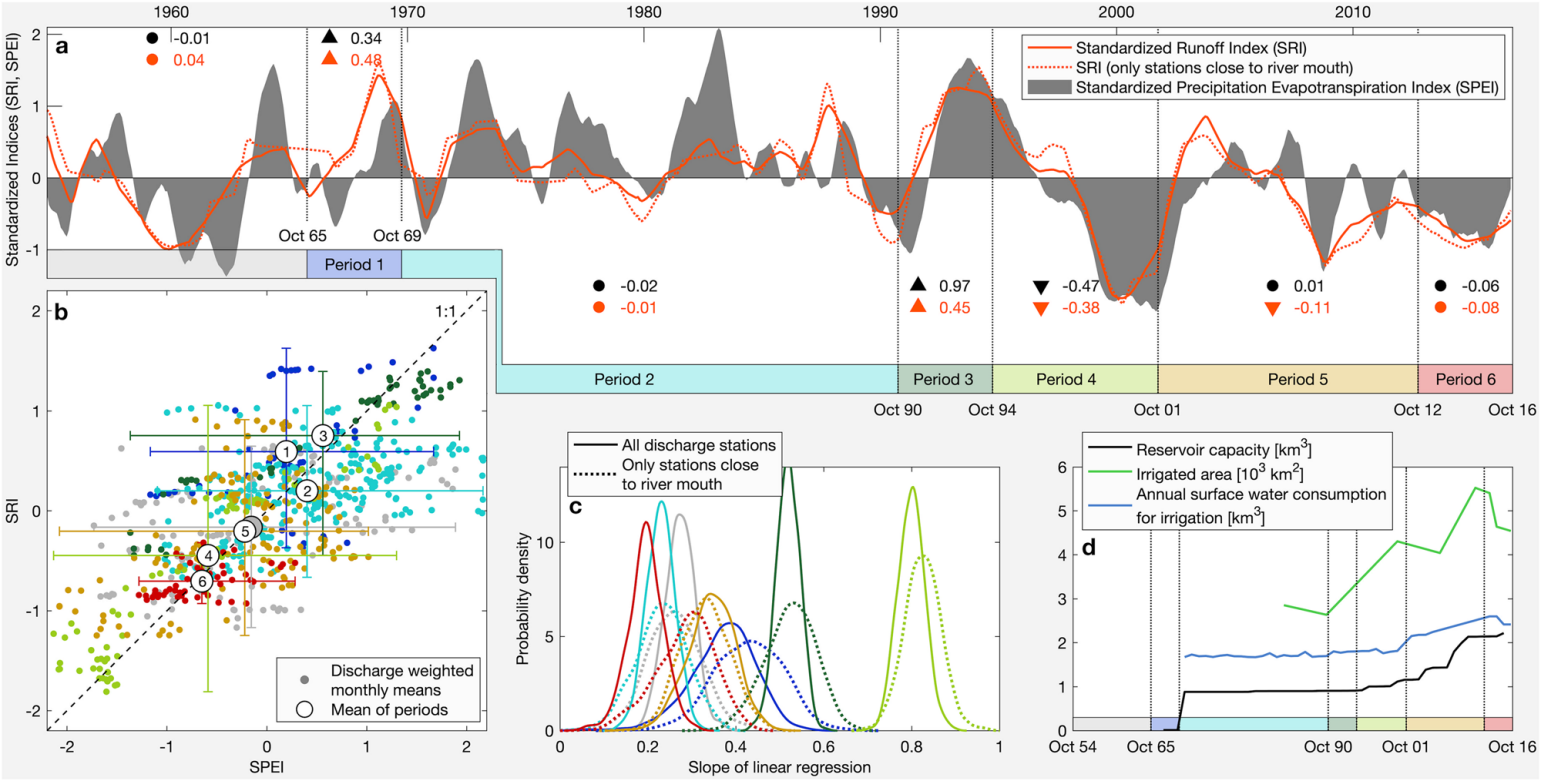
Statistical analysis of runoff and weather time series. (**a**) Temporal evolution of 12-months SPEI and SRI (moving average filter with a kernel size of 12 months). A pointing up and down triangle shows a significant positive and negative Mann-Kendall trend, respectively. A dot means no significant trend (p = 0.05). (**b**) Relationship between discharge weighted monthly mean SPEI and SRI values. (**c**) Probability density plot of the linear regression slope of the SRI-SPEI relationship based on bootstrap sampling. (**d**) Temporal evolution of anthropogenic influences affecting Lake Urmia’s water budget. (**a**–**d**) The color code used refers to the periods defined in Figs. 2 and 3a and grey colored lines or dots representing the period 1954–1965. (Plots are generated using MATLAB R2019b, www.mathworks.com).

Precipitation and evaporation are the principal natural boundary conditions and drivers for streamflow. Here, we compare the temporal patterns of precipitation and evaporation with the discharge volumes of the rivers. To allow for a direct comparison, we applied the 12-months Standardized Precipitation Evaporation Index (SPEI), which is a multi-scalar measure accounting for precipitation and potential evaporation^34^. In analogy to the SPEI, we used the Standardized Runoff Index (SRI)^35^ representing the river flows. Both indices have the same scale and positive values indicating wetness, while negative values indicate dryness. For the SRI two different time series are used: one, which is based on the discharge weighted mean of all discharge stations and another one, which only bases on those stations that are the closest for each main river to the river mouth (Fig. 3a). The latter SRI series represents the inflow reaching the lake and is more relevant for the lake water budget. In analogy to the six periods, defined earlier for the evolution of the lake volume, we have also subdivided these time series into six periods. In addition to the six periods, we also show statistical parameters for a period from 1954 to 1965. Here it should be noted, however, that the corresponding data set might be less robust due to less operating hydrometric stations described above.

The SRI seems to follow the SPEI with a good approximation, which is also illustrated by the Mann-Kendall trends. Only in period 5 the trends differ significantly, with no significant trend for SPEI and a negative trend for SRI (Fig. 3a). Interestingly, this period is also characterized by a strongly increasing capacity of reservoirs in the catchment, i.e. it almost doubled from 1.2 km^3^ to 2.1 km^3^, and an increasing surface water consumption for irrigated agriculture (Fig. 3d). To further analyze the relationship between SPEI and SRI, their individual monthly values are compared (Fig. 3b). As expected, during periods which show a significant decline in lake volume (periods 4 and 5) or a very low volume (period 6), mean SRI and SPEI values are negative. During periods 1 and 3, Lake Urmia experienced an increase in water level, which is reflected by positive values of mean SRI and SPEI. Moreover, it can be observed that during period 1, the mean SRI is higher than the SPEI, which indicates more favorable discharge conditions. The lowest mean SRI in relation to the corresponding SPEI can be found in period 2. Other mean SRI and SPEI differ less, i.e. they plot quite close to the 1:1 line, including in periods 4 and 5 during which the severe lake level drop happened (Fig. 3b). We also analyze the slope of the linear regression of the monthly SPEIs and the responding SRIs. For this purpose, we not only analyze a single slope, but perform a bootstrap sampling with 1,000 data samples per period. The result of the bootstrapping analysis is displayed by a probability density function of the slopes for each period (Fig. 3c). Generally, higher slope values indicate stronger responses of river flow to changes in weather. Interestingly, anthropogenic alteration can cause both a weakened and an amplified response. A weakened response is usually expected due to damming, while an amplified response often results from water extraction for irrigation. This amplification commonly results from an exceptionally high irrigation water withdrawal during dry seasons and a reduced extraction during wet seasons^36^. In our case, the strongest response appears for period 4, during which the most rapid lake level decline happened (Figs. 2 and 3c). Based on the previous discussions, this might be a consequence of increased extraction of irrigation water to mitigate the impact of drought. However, this explanation seems not very likely as the majority of the very dry months (very low SRI and SPEI) plotting well above the 1:1 line (Fig. 3b), i.e. they don’t show exceptionally low discharge rates related to the weather conditions. In contrast, the weakest response (lowest slopes) of SRI to SPEI occurred during periods 2 and 6 followed by period 1 and 5 (Fig. 3c). In this case, too, the explanation that the dams could be the cause seems rather unlikely. Since the number and likewise the cumulative volume of dams has steadily increased over time (Fig. 3d), a steady increase in influence (i.e. weakening of response) could have been expected.

Results show that the temporal pattern of river flow rates can be well explained by weather changes, while an anthropogenic impact is not so obvious. However, in our analyses we have so far focused on an explanation for the temporal variability of the river runoff, which could imply the risk of a systematic influence being ignored. And indeed, a look at the temporal evolution of surface water consumption for irrigation reveals such a systematic influence (Fig. 3d). Since about 1970 irrigation was promoted for agricultural development and first large-scale water management was introduced with the construction the reservoirs Bukan and Mahabad (Fig. 1a)^37^.

Since then, records of surface water extraction for irrigation show high, but relatively constant rates. The only remarkable change occurred during the period 5 with an increase from 2.1 to 2.6 km^3^a^−1^. During the extreme lake level drop in period 4, the increase of irrigation water extraction was only about 0.2 km^3^a^−1^, while the inflow to the lake decreased by 90% from 6.0 to 0.6 km^3^a^−1^ (Figs. 2 and 3d). However, it is important to note that the withdrawal rates are generally on a quite high level and often (especially in the recent years) exceed the remaining inflow into the lake. On the basis of this perception and previous results, it can be concluded that although the massive irrigation withdrawal of surface water does not seem to directly cause the significant decline in the lake level over the last two decades, it has greatly weakened the lake’s resilience, making it vulnerable to climate change.

This hypothesis is also supported by a parsimonious modeling experiment (Supplementary Information 1). Here it could be shown that even under more or less natural inflow conditions (irrigation water extraction added to the inflow), the simulated lake volume also decreased significantly over the last two decades. Its temporal variability does not differ too much from the observed lake volume either. However, under these natural inflow conditions, the simulated lake volume is constantly well above the simulated one, which takes irrigation water abstraction into account. Hence, the volume decrease of the lake under natural inflow conditions is much less pronounced, i.e. for the year 2017 the simulated lake volume without irrigation water extraction (8.3 km^3^) is more than four times larger than the volume with irrigation water extraction (1.9 km^3^, Supplementary Fig. S1).

### Reservoirs and discharge

From 1967 to 2015, 57 reservoirs with total capacity of 2.2 km^3^ were built in the Lake Urmia catchment to ensure a stable water supply for irrigated agriculture. An analysis of the impact of these reservoirs on the river runoff (Supplementary Information 2) revealed that runoff time series from downstream hydrometric stations do not show negative runoff trends more frequently than those that are not influenced, i.e. before reservoir construction or upstream of a reservoir (Supplementary Fig. S2). After the construction of the reservoirs, on the other hand, the discharge is reduced on average by about 10%. Here, however, it is difficult to say whether this is a direct consequence of reservoir construction or merely a consequence of the generally declining runoff of recent decades. A clear difference between the runoff time series before and after reservoir construction is the interannual variability. After reservoir construction, the interannual variability is more balanced, i.e. runoff is relatively higher during dry months and relatively lower during wet months compared to the temporal runoff pattern before reservoir construction (Supplementary Fig. S3). This shows quite clearly the intended water retention to support the irrigation water supply in the dry months.

### Implications on future perspectives

**Figure 4:**
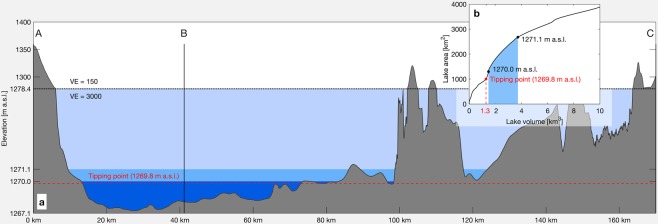
Morphology of Lake Urmia. (**a**) Cross section of Lake Urmia with a vertical exaggeration (VE) of 150 and 3000 above and below 1278.4 m a.s.l., respectively. The location of the cross section is displayed in Fig. 1. (**b**) Volume-area relationship; the blue area shows the range of monthly values between 2013 and 2018. (Plots are generated using MATLAB R2019b, www.mathworks.com).

Evaporation from the lake strongly depends on the lake surface area and its salt concentration and thus counteracts low inflow rates. However, this only works effectively as long as there is a strong reduction of the lake area as a feedback to a decreasing volume. To analyze this, we need to take a look at the lake morphology. Lake Urmia is generally shallow and has a maximum depth of only about 10 meters. This shallowness is also reflected by a quite high average area-volume ratio of about 900 km^−1^ (considering a lake level range of 1267.2–1278.4 m a.s.l.). In comparison, the Great Lakes in the US having an average area-volume ratio of about 10 km^−1^ ^38^. In particular in the south, the lake bottom’s marginal planes are flat and have extremely gentle slopes. On the other hand, the northern part of the lake is slightly deeper (Fig. 4a). This morphology leads to a very non-linear volume- area relationship. A strong reaction of the lake area on a reduced volume can be observed for a lake volume between 1.3 and about 4 km^3^, while above and below this volume the reaction is weaker (Fig. 4b). The lower tipping point at about 1.3 km^3^ (sharp change in slope, Fig. 4b) is reached when the shallow southern part of the lake falls completely dry. The lake volume has fluctuated just above this tipping point since 2013, where the strongest reaction of the lake area on volume changes can be observed. This may have contributed to the relatively stable lake volume of recent years, although the SPEI index was negative and despite the growing significance of agricultural water extraction. However, it should be noted that exceeding this tipping point leads to a reduced buffer effect and thus to a loss in resilience to climatic changes and agricultural water use.

**Figure 5:**
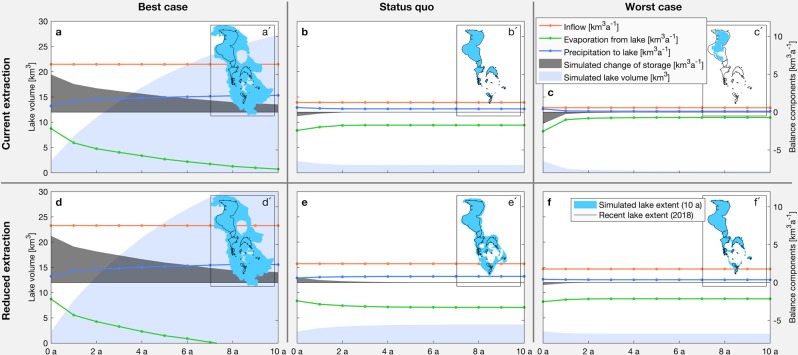
Development scenarios for Lake Urmia. Climatic best-case (mean precipitation, potential evaporation and inflow of period 3), status quo (mean of period 6) and worst-case (mean of seasons 1999/00 to 2000/01) scenarios. (**a**–**c**) Scenarios assuming current irrigation water extraction. (**d**–**f**) Scenarios assuming a reduced irrigation water extraction by 1.2 km^3^a^−1^ (50% of current surface water extraction for irrigation). (Plots and maps are generated using MATLAB R2019b, www.mathworks.com).

To draw some conclusions for Lake Urmia’s future development, we run a series of forward simulations based on the lake water balance. The forecasting period of these simulations is ten years and six different scenarios are analyzed. First, we distinguish between a best-case, status quo, and worst-case scenario for the climatic boundary conditions (Fig. 5a–c). These scenarios base on observed mean precipitation, potential evaporation and inflow rates, for the period 3, period 6 and the two hydrological years from October 1999 to September 2001, respectively (Table 1). Here, the latter one was chosen due to exceptionally low inflow rates and the most severe drop in lake volume (Fig. 2). In addition, for these three climatic scenarios, we assumed an increased inflow of 1.2 km³a^−1^ into the lake, due to water savings in the agriculture sector (Fig. 5d–f). This corresponds to 50% of the surface water extraction for irrigation in 2016, savings that are considered possible in related studies^1,28^.

**Table 1:** Climatic boundary conditions.

Scenario	Related time period	Precipitation [mma^−1^]	Potential evaporation [mma^−1^]	Inflow [km^3^a^−1^]
Best-case	Period 3	414	1606	6.3
Status quo (current conditions)	Period 6	303	1776	1.3
Worst-case	Oct 1999 – Sep 2001	213	1860	0.6

Climatic boundary conditions for the simulations of development scenarios.

Our analysis show that for the climatic best-case scenario, the lake could reach a volume of 15 km^3^, which corresponds to an area of 4400 km^2^, already after about three years, even if agricultural withdrawal is not reduced (Fig. 5a,d). The gain in volume is significantly higher compared to observations in the best-case climatic reference period, due to the initially much smaller lake area, resulting in lower evaporation. However, the climatic status quo and also the worst-case scenarios are more likely, based on regional climate projections^39,40^. Here, agricultural water savings would have a significant impact on the lake volume. While without water savings the lake volume would further shrink (status quo scenario, Fig. 5b) or even dramatically shrink (worst-case scenario, Fig. 5c), a 50% reduction in withdrawal would lead to a 60% increase in lake volume after four years in the status quo scenario (Fig. 5e), and at least to a more or less constant lake volume, compared to today, even for the worst-case scenario (Fig. 5f).

## Conclusion

We could show that in the last decades variation in the volume of Lake Urmia was mainly triggered by changes in climatic conditions, and even without agricultural water extraction the general trend of the lake volume variations would have been the same. However, this conclusion does not mean that human influence on the hydrological system in the Lake Urmia catchment is negligible. Using a parsimonious modeling experiment, we were also able to show that agricultural extraction has a massive influence on the resilience of the lake, as it exaggerates the general trend of declining lake volume, especially in the last two decades. This means, that without agricultural extraction the lake volume would also have decreased significantly in the last two decades, but would still have ended up at a much higher volume. Interestingly, the specific morphology of the lake could, to some extend buffer reduced inflows. However, the current climatic conditions together with the retraction of the lake to its northern and slightly deeper parts brought it into a very critical and labile state, close to the tipping point where it loses its ability to buffer reduced inflows by a reduced surface area. As agricultural water withdrawals are under the current climatic conditions comparable to the remaining surface water inflow volumes, or even higher, any changes in water withdrawal would have a significant impact on the lake volume. This is a risk, as well as an opportunity. Maintaining or even increasing the current extraction rates could result in a complete collapse of the lake, especially if climate would further get dryer. However, substantial but realistic agricultural water savings could stabilize the lake by keeping the lake volume above the crucial tipping point, bringing therefore back its ability to buffer, even if climate would get dryer. Considering the current climatic conditions, it could as well lead to a significant volume and surface area increase of the lake, thus regaining its role as a very precious and special ecosystem.

## Methods

### Data

The lake volume evolution as well as the quantification and further analysis of the different water balance components (inflow, precipitation, evaporation and change of storage) base on a series of different data sets. These include: (**i**) mean monthly and annual lake level for the period 1965–2018 and 1931–2017, respectively (Iran Water Resources Management Company); (**ii**) monthly and annual inflow into the lake for the period 1965–2016 and 1953–2017, respectively (Ministry of Energy); (**iii**) monthly river runoff for a total of 132 gauging stations with records between 1952 and 2016 (Ministry of Energy); (**iv**) monthly potential evaporation, calculated based on routine weather data using a simplified version of the Penman equation^41^, for Tabriz from 1952 to 2017 (Iran Meteorological Organization); (**v**) monthly precipitation for the stations Tabriz (1951–2017), Urmia (1951–2017), Sahand (1985–2017), Mahabad (1985–2017) and Miandoab (2002–2017) (Iran Meteorological Organization); (**vi**) monthly river water extraction for irrigation from 1970 to 2016 (Ministry of Agriculture); (**vii**) evolution of total area for irrigation agriculture in the Lake Urmia catchment between 1984 and 2016 (Ministry of Agriculture); (**viii**) information about the 41 principal reservoirs in the catchment, i.e. construction date and capacity (Ministry of Energy); (**ix**) bathymetry of Lake Urmia with a resolution of 30 × 30 m, surveyed in 2017 by an echo sounding (50–200 kHz) mapping campaign (by the Ministry of Energy’s Water Research Institute on request of Urmia Lake Restauration Program).

### Level-area-volume relationship

The lake area and volume are calculated based on the lake level and the raster data set of the bathymetry. To calculate the area, the number of pixels, which are equal or lower than the corresponding lake level, is multiplied with the pixel size (900 m^2^). Subsequently, the mean depth is calculated by subtracting the mean elevation of the pixels, which are equal or lower than the lake level, from the lake level. Multiplying the mean depth with the area results the lake volume.

### Salt water evaporation

Evaporation from saline water bodies depends on meteorological variables, but also on the salinity and the ionic composition. As the salinity increases, the free energy of the water molecules is reduced, resulting in a decrease in the saturation vapor pressure above the water surface and thus a decrease in evaporation^42,43^. Here, the relationship of actual salt water evaporation (E_sal_) to freshwater evaporation (E_fresh_) can be represented by the salinity dependent empirical ratio α^42,43^, Eq. (2).

2 $$\alpha =\frac{{E}_{sal}}{{E}_{fresh}}$$

**Figure 6:**
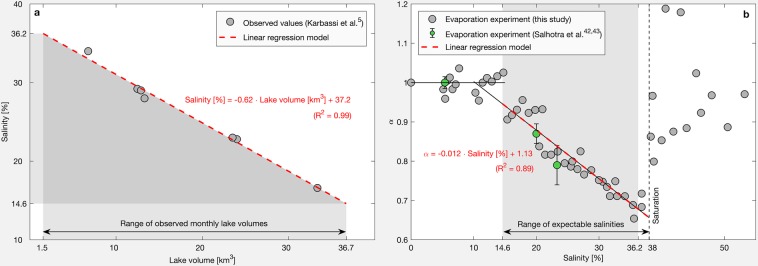
Impact of lake volume on evaporation. (**a**) Relationship of salinity and lake volume^5^. (**b**) Relationship of the empirical ratio α and salinity; error bars for α from the evaporation experiment of Salhotra *et al*.^42,43^ show the standard deviations for a range (n = 24) of different temperatures (16–35 °C) and humidities (29–62%). (Plots are generated using MATLAB R2019b, www.mathworks.com).

Lake Urmia is an endorheic basin and therefore the salt concentration depends strongly on the lake volume. A linear regression model, based on a number of observations^5^, explains this dependence of the salinity on the lake volume quite well (Fig. 6a). In order to determine the empirical ratio α, we performed evaporation experiments for eight different salt solutions and a fresh water reference. The ionic composition of the salt solution used was similar to that of Lake Urmia^5^ and the initial salt concentrations ranged from 5% to 38%. During the experiment, temperature and relative humidity were kept constant at 30 °C and 20%, respectively, by means of an environmental chamber. The experiment lasted 7 days and each day the evaporation loss was measured gravimetrically. For the expected concentration range, based on the range of observed volumes (Fig. 6a), a linear regression model is used to describe the relationship between α and salinity (Fig. 6b). Subsequently, actual salt water evaporation rates for Lake Urmia were estimated by multiplying potential evaporation rates with the corresponding value for α, which was derived from the introduced linear models.

It should be noted that this approach constitutes a simplification. Actually, α not only depends on the salinity, but it is also influenced by meteorological variables. Two studies of Salhotra *et al*.^42,43^ have analyzed this influence. They experimentally determined α for a range (n = 24) of different temperatures (16–35 °C) and humidities (29–62%) for three concentrated Mediterranean Sea water samples with about 5.4%, 20%, and 23.3% salinity. Resulting values for α showed an average standard deviation of 0.06, which could be seen as a first approximation for the uncertainty of the presented approach. Being aware of the existence of more accurate concepts, we still consider the applied approach appropriate as it does not require additional and, in our case, not continuously available data such as water temperature and humidity directly above the lake surface.

### Statistics

The 12-months standardized precipitation evaporation index (SPEI)^34^ and the 12-months standardized runoff index (SRI)^35^ are calculated individually for all 132 runoff stations^44^. Here, for the SPEI the closest available weather station, based on Thiessen polygons, is used. Subsequently, for each month during the test period from 1965 to 2016, the runoff-weighted averages for the indices are calculated (Fig. 3a). In order to investigate the temporal development of both time series, they are split into the six periods, which characterize the general trends in lake volume evolution (Fig. 2). For each of these periods and for both indices a seasonal Mann-Kendall test is performed and the Sens slope is calculated^45,46^. The significance level for the seasonal Mann-Kendall test is 0.01 and the starting month for the 12-months interval is October (start of the hydrological year). Bootstrap sampling of the slope of the simple linear regression model of the SRI-SPEI relationship for the six periods is performed with a number of 1000 data samples (Fig. 3c).

## Supplementary information

Supplementary Information.

## Data Availability

All data used in this study is available from the corresponding author upon reasonable request.

## Code availability

Matlab codes are archived at: https://github.com/stephan-schulz-1/urmia.git.
