# Supplementary material for: Climate change or irrigated agriculture – what drives the water level decline of Lake Urmia
Source: Sci Rep. 2020 Jan 14;10:236. doi: 10.1038/s41598-019-57150-y (PMC6959231; doi:10.1038/s41598-019-57150-y)
Supplement: Supplementary file 1 — Supplementary Information. [file 41598_2019_57150_MOESM1_ESM.pdf]

Supplementary Information

Climate change or irrigated agriculture – what drives the water level decline of Lake Urmia

Stephan Schulz[1,*], Sahand Darehshouri[1], Elmira Hassanzadeh[2], Massoud Tajrishy[3], Christoph Schüth[1]

[1] Technische Universität Darmstadt, Institute of Applied Geosciences, Schnittspahnstr. 9, 64287 Darmstadt, Germany

[2] Polytechnique Montréal – Department of Civil, Geological and Mining Engineering

[3] Sharif University of Technology, Urmia Lake Restoration Program, Department of Civil Engineering, Azadi Ave, P.O.Box: 11155, 9313 Tehran, Iran

[*] Corresponding author: Stephan Schulz, e-mail: schulz@geo.tu-darmstadt.de, ORCID ID: https://orcid.org/0000-0001-7060-7690

Supplementary Information 1

Based on the water balance and the computation of its individual components, we carried out two simulations to analyze the influence of surface water extraction for irrigation agriculture on the evolution of the lake volume. For the first scenario we simulated the change of the lake volume depending on the observed climatic boundary conditions and the observed inflow rates ("Simulated lake volume", Fig. S1). For the second scenario we added the irrigation water extraction to the inflow to mimic natural runoff conditions ("Simulated lake volume, no irrigation", Fig. S1). Limited by the availability of data on surface water extraction, the simulations were carried out for a period from 1971 to 2017. The common starting point for the lake volume is the observed volume of 23.9 km^3 in 1971. For the year 2017 the simulated lake volume without irrigation water extraction (8.3 km^3) is more than four times larger than the volume with irrigation water extraction (1.9 km^3).

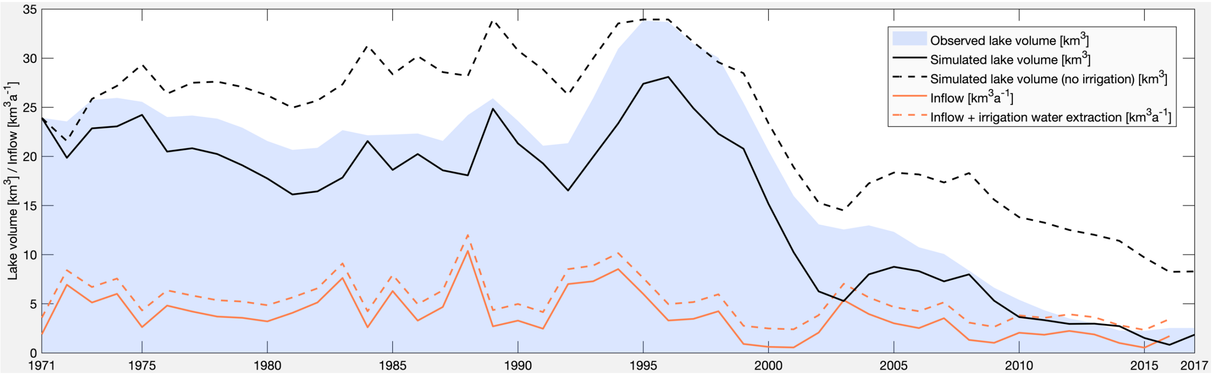

Fig. S1 | Simulated lake volume evolution. Simulated lake volume evolution from 1971 to 2017 for observed inflow rates and natural inflow rates (inflow + irrigation water extraction). (Plot is generated using MATLAB R2019b, www.mathworks.com.)

Supplementary Information 2

To analyze the impact of the reservoirs on the temporal evolution of river discharge, we subdivided the discharge time series in those ones, which are not influenced by dams (i.e. before reservoir construction or upstream of a reservoir (Fig. S2a), and in those ones, originating from a station located downstream of a reservoir (Fig. S2b). Subsequently, we compared the Mann-Kendall trends and the mean discharge rates of both time series sets. The somehow surprising result is that there is not a big difference. Time series from stations located downstream of a reservoir show even less often negative trends (32%) compared to those, which are not influenced by dams (54%, Fig. S2). In order to compare

the difference of the mean discharge rates, we could only use stations (n = 30), which are located downstream of a reservoir and have discharge records before as well as after construction. The runoff-weighted mean discharge after storage construction is about 10% lower than before construction.

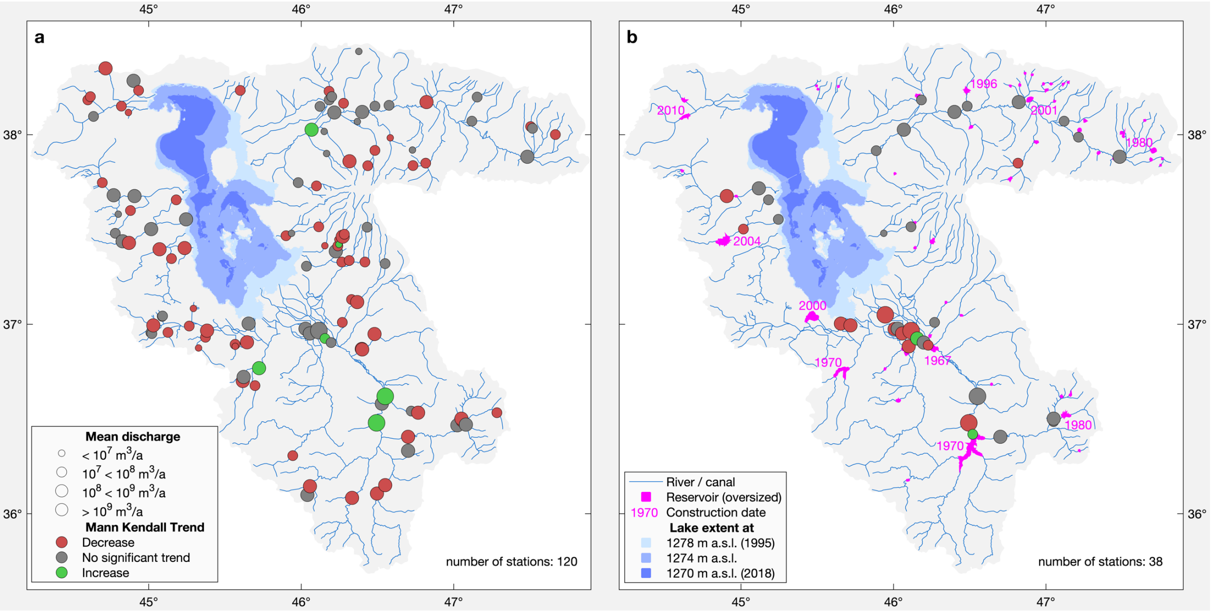

Fig. S2 | Mann-Kendall trends and mean annual flow rates for discharge stations. a, Upstream of reservoirs or before reservoir construction. **b**, Downstream of operating reservoirs. (Maps are generated using MATLAB R2019b, www.mathworks.com.)

The separation of the runoff time series into those influenced by reservoirs and those not influenced (see above) also served to analyze the interannual temporal runoff behavior. Mean runoff time series are normalized to the maximum discharge occurring in May for both cases (Fig. S3).

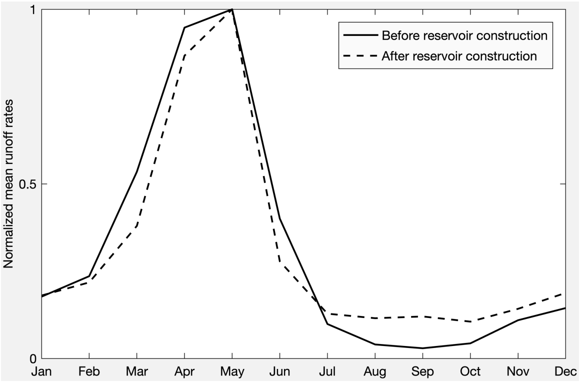

Fig. S3 | Interannual runoff variability. Interannual runoff variability of normalized mean runoff rates before and after dam construction. (Plot is generated using MATLAB R2019b, www.mathworks.com.)
